# Polysorbate 80 Differentially Impacts Erinacine Production Profiles in Submerged Cultures of *Hericium*

**DOI:** 10.3390/molecules30132823

**Published:** 2025-06-30

**Authors:** Abigail Veronica Smith, Honghui Zhu, Lili Mats, Gale Bozzo

**Affiliations:** 1 Department of Plant Agriculture, University of Guelph, 50 Stone Road East, Guelph, ON N1G 2W1, Canada; asmith85@uoguelph.ca; 2 Guelph Research and Development Centre, Agriculture and Agri-Food Canada, 93 Stone Road West, Guelph, ON N1G 5C9, Canada; honghui.zhu@agr.gc.ca (H.Z.); lili.mats@agr.gc.ca (L.M.)

**Keywords:** erinacine, *Hericium americanum*, *Hericium coralloides*, *Hericium erinaceus*, mycelium, polysorbate 80

## Abstract

The mycelia of *Hericium erinaceus* contain neuroprotective cyathane diterpenoids (e.g., erinacine A). There is evidence that cultivation of submerged mycelia with surfactants increases glucose uptake and biomass, but the impact on erinacine production is unknown. Here, we tested the impact of glucose and polysorbate 80 on the mycelial erinacine profiles of five *Hericium* strains cultivated under submergence, including those of *Hericium erinaceus*, *Hericium americanum*, and *Hericium coralloides*. Metabolite profiling confirmed that mycelial extracts contained 13% to 91% of the erinacines A, C and P in additive-free cultures of all strains, with the remainder secreted to the culture medium. Overall, erinacine P production was several orders of magnitude greater than that of the other erinacines, except for *H. erinaceus* (DAOMC 251029), where erinacine C was most evident. *H. coralloides* (DAOMC 251017) produced the greatest concentrations of erinacines A and P. For the most part mycelial erinacine concentrations were reduced in cultures co-supplemented with glucose and polysorbate 80. This treatment caused an 83–100% reduction in the concentrations of erinacines A, C, and P in the mycelial extracts of most strains. By contrast, there was evidence that glucose and polysorbate 80 had no effect on erinacine A production within mycelia of *H. americanum*, and erinacine P concentrations in *H. erinaceus* (DAOMC 251029) and *H. americanum* (DAOMC 251011). In most strains, the secretion of erinacines to the culture medium declined with glucose and polysorbate 80. Conversely, these additives increased the concentrations of erinacines C and P in the culture medium filtrate of *H. americanum* (DAOMC 21467) and yielded more secreted erinacine P in *H. erinaceus* (DAOMC 251029). The information provides feasible strategies to produce mycelia with unique erinacine profiles including those rich in erinacine P.

## 1. Introduction

The genus *Hericium* consists of basidiomycetes that develop on decaying wood, including the culinary mushroom lion’s mane (*Hericium erinaceus* (Bull.) Pers.). Lion’s mane mushroom is used in traditional Chinese medicine for gastrointestinal issues and neurological ailments [1,2]. Although not as widely studied as *H. erinaceus*, *Hericium americanum* Ginns (bear’s head tooth mushroom) and *Hericium coralloides* (Scop.) Pers. (coral tooth mushroom) are naturally distributed in North America and Europe [3,4,5]. A phylogenetic comparison of the sequences corresponding to the internal transcribed spacer (ITS) region of nuclear ribosomal DNA has revealed that these two species are closely related to *H. erinaceus* [3]. The potential medicinal relevance of *H. americanum* and *H. coralloides* requires further information on their bioactive constituents.

**Figure 1:**
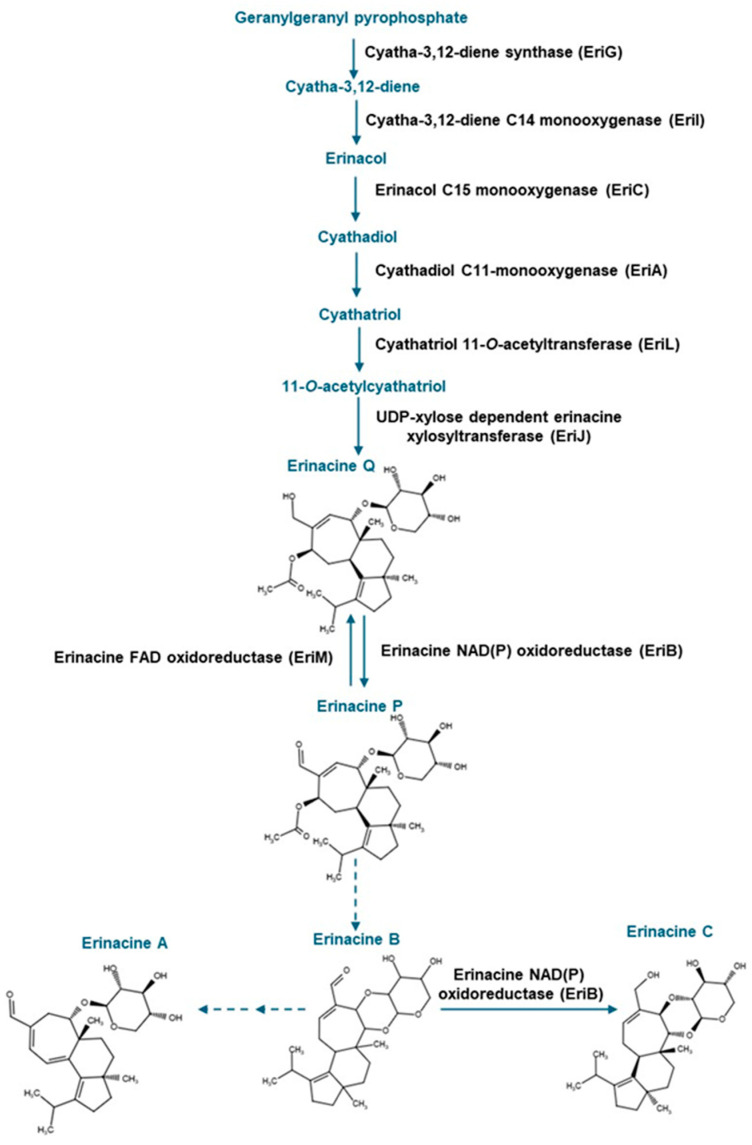
Metabolic steps in the erinacine biosynthesis pathway involved in the production of erinacines A and C and their shared precursor erinacine P. The scheme was adapted from Doar et al. [13], and steps as described in MetaCyc (https://biocyc.org/pathway?orgid=META&id=PWY-8408 (accessed on 8 May 2025)). Solid blue arrows and black text represent known enzymatic steps; dashed blue arrows represent uncharacterized metabolic steps. Erinacine structures were drawn with Chemical Sketch Tool (https://www.rcsb.org/chemical-sketch (accessed on 8 May 2025)).

To date, it is known that the mycelia of *H. erinaceus* are rich in cyathane diterpenoids known as erinacines, most of which occur as xylosides. Nearly 20 different erinacines occur in *H. erinaceus*. The pharmacological value has been established for a few of these compounds, including the widely studied erinacine A [6,7]. To date, some of these erinacines have been found to occur in *Hericium flagellum* (also known as *H. alpestre*) [8], but their distribution in other *Hericium* spp. is not established. Previous research including studies using rodent models point to the role of erinacine A in minimizing oxidative stress-related processes, including inflammation associated with neurological disorders such as Parkinson’s disease [6,9]. Moreover, neurocognitive abilities can be improved in early Alzheimer’s disease patients following the administration of tablets containing *H. erinaceus* mycelia enriched in erinacine A [9]. Erinacine C also occurs in *H. erinaceus* mycelia [10]. In vitro analyses revealed that erinacine C induces neurotrophic activity in neuronal astrocytic cells [11]. Moreover, erinacine C reduces inflammation associated with traumatic brain injury [12]. It has been proposed that both erinacines A, B and C are all derived from the metabolic precursor erinacine P [13,14] (Figure 1).

*H. erinaceus* mycelia can be cultivated in shake-flask cultures under liquid submergence [15]. Recent evidence demonstrates that the mycelia of *H. americanum* and *H. coralloides* can also be produced in shake-flask cultures [16]. The production of erinacine A in submerged mycelia cultures varies across *H. erinaceus* strains/isolates [17]. Similarly, erinacines C and P are produced within and secreted from submerged *H. erinaceus* mycelia grown in shake-flask cultures [10,18]. It is worth noting that mycelial biomass and erinacine A production of *H. erinaceus* are both elevated when the basal growth medium is supplemented with additional glucose during cultivation under submergence [15,19]. To date, there is no knowledge of whether erinacines, including erinacines A, C, and P, occur in *Hericium* spp. other than *H. erinaceus* and *H. flagellum*.

The addition of non-ionic surfactants (i.e., polysorbate 80) to the liquid culture medium promotes the uptake of glucose and increases biomass yield in the mycelia of *Pleurotus tuber-regium* [20]. Some of these phenomena have been reported for the liquid cultivation of mycelia belonging to other fungal species, including *Ganoderma lucidum* [21] and *Inonotus obliquus* [22]. The addition of Tween 80 (a polysorbate 80 product) to the fermentation broth increases glucose acquisition, biomass accumulation and β-glucan production in *H. erinaceus* mycelia [23]. Tween 80 also impacts biomass yield and polysaccharide production in the mycelial cultures of other fungi such as *Cordyceps sinensis* and *Lentinus tigrinus* [24,25,26]. It is worth noting that Okumura et al. [23] determined the Tween 80 yielded greater biomass of liquid culture-grown *H. erinaceus* mycelia when supplied at final concentrations in the range of 0.5 to 4% (*w*/*v*), as opposed to cultures supplemented with Tween 20, Tween 40 or Tween 60. Moreover, polysorbate 80 has little toxicity in fungal cultures as compared to other surfactants such as sodium dodecyl sulphate and tetradecyltrimethylammonium bromide [27]. Although polysorbate 80 increases glucose uptake and polysaccharide production in mycelial cultures, it is unknown whether this surfactant impacts other glucose-derived metabolic processes in submerged *Hericium* cultures such as erinacine production. This seems plausible given that erinacines are diterpenoids, and in fungi these metabolites are derived from the mevalonate pathway, which requires an input of acetyl CoA [28]. Acetyl CoA is formed during the respiration of glucose. The overall aim of this study was to determine the impact of glucose supplementation with and without the addition of polysorbate 80 on erinacine production in *Hericium* spp., including the lesser-explored *H. americanum* and *H. coralloides*.

## 2. Results and Discussion

### 2.1. Effect of the Addition of Polysorbate 80 to Shake-Flask Cultures on the Biomass of Hericium Mycelia

**Figure 2:**
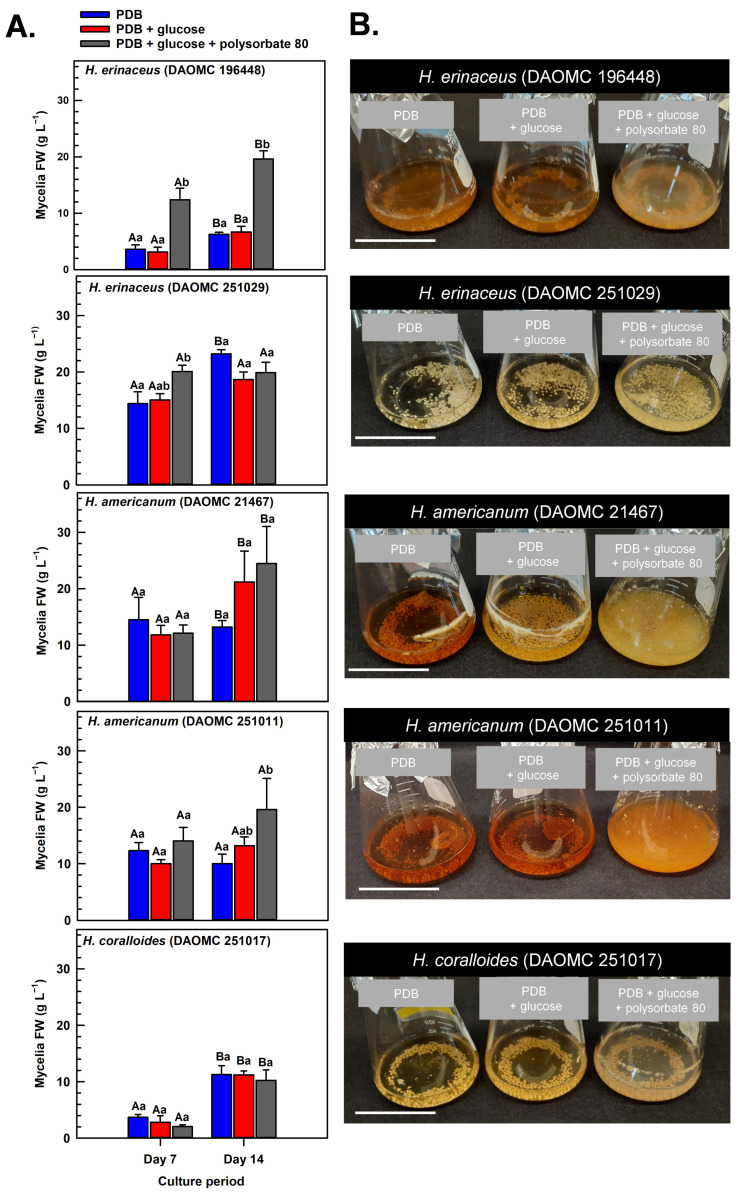
(**A**) Alterations in the fresh weight (FW) of shake-flask cultures of *Hericium* spp. mycelia grown on potato dextrose broth (PDB) for up to 14 days. Within each plot, blue-filled bars, red-filled bars, and grey-filled bars represent the respective FW of mycelia cultivated on PDB, PDB supplemented with 2% (*w*/*v*) glucose, and PDB supplemented with 2% (*w*/*v*) glucose and 1% (*w*/*v*) polysorbate 80. Each datum represents the mean FW ± SE of three separate experimental replicates. Within each plot, uppercase letters represent statistical comparisons within a cultivation treatment across the culture period. Lowercase letters represent statistical comparisons across the cultivation treatments at each culture period sampling time. Means sharing the same letter are not statistically different (*p* ≤ 0.05). (**B**) Representative images of shake-flask cultures of *Hericium* spp. mycelia after 14 days of cultivation on PDB (left-side flask in each image), PDB with 2% (*w*/*v*) glucose (center flask in each image), and PDB with 2% (*w*/*v*) glucose and 1% (*w*/*v*) polysorbate 80 (right-side flask in each image). The white scale bar line at the bottom left corner of each image is equivalent to 5 cm.

Submerged mycelia of various *Hericium* strains were cultivated in shake-flask liquid cultures for up to 14 days. The cultures were supplied with potato dextrose broth (PDB), a medium previously used for the cultivation of *H. erinaceus* and related species [5,29,30], including shake-flask cultures of *H. coralloides* [31]. Prior research has shown that glucose (i.e., initial concentration of 3% *w*/*v*) is fully depleted within 8 days of its supply to shake-flask cultures of *H. erinaceus*, and that polysorbate-based surfactants promote glucose uptake and mycelial growth [20,21,22,23]. Here, we investigated the impact of supplementing shake-flask cultures with glucose and polysorbate 80 on the dynamics of mycelial growth of two different *H. americanum* strains, two *H. erinaceus* strains, and a single *H. coralloides* strain (Figure 2). These strains were from the Canadian Collection of Fungal Cultures (annotated as DAOMC). All cultures that were supplied with glucose at the beginning of the culture period were resupplied with 2% (*w*/*v*) glucose 8 days thereafter. In cultures containing PDB without additives (i.e., control), the total culture fresh weight (FW) varied across the *Hericium* strains by the end of the 14-day cultivation period. With respect to the control treatment, the greatest mycelia FW was apparent in 14-day old *H. erinaceus* (DAOMC 251029). This strain accumulated 76 to 270% more biomass than the other *Hericium* strains. These variable growth rates fit with previous research demonstrating a wide range of mycelial biomass yields across various *Hericium* spp. cultivated on potato dextrose agar (PDA) for 10 to 15 days [29,30]. For most strains and their cultivation treatments, the maximum mycelia FW occurred on day 14 of growth in the shake-flask system. Conversely, maximum biomass was realized 7 days earlier for *H. americanum* (DAOMC 251011) and for all glucose-containing *H. erinaceus* (DAOMC 251029) cultures.

Krzyczkowski et al. [15] reported a near 4-fold increase in mycelial biomass in shake-flask cultures of *H. erinaceus* when basal salt medium was supplemented with glucose. Conversely, the growth dynamics of all five *Hericium* strains investigated here were not altered by the supplemental 2% (*w*/*v*) glucose treatment relative to cultures containing PDB alone. It is worth noting that PDB consists of 2.4% (*w*/*v*) dextrose, which is chemically similar to glucose. Thus, all glucose supplemented cultures had 4.4% (*w*/*v*) glucose/dextrose molecules. Our findings are consistent with the comparable mycelia biomass of submerged *H. erinaceus* mycelial cultures supplied with initial glucose concentrations in the range of 2 to 4% (*w*/*v*) [19]. Alternatively, the absence of an increase in biomass yield with supplemental glucose alone could be due to restricted oxygen diffusion and metabolite uptake into the mycelial pellets that are formed with liquid submergence [32], which are more pronounced in larger pellets (i.e., >2 mm) [33]. Thus, it is likely that hypoxic conditions occur at the center of the pellet leading to a reduction in aerobic respiration and, hence, a decline in ATP production required for growth. In fact, a transcriptomic analysis of *Cordyceps militaris* mycelia revealed the accumulation of fermentation-related gene transcripts in response to prolonged liquid submergence [34].

Previous research has shown that surfactant additives increase glucose uptake and hence growth of submerged mycelia [22,23]. To a similar extent, a large increase in mycelial FW was evident for some of the *Hericium* strains co-treated with glucose and polysorbate 80 relative to the cultures grown without additives (Figure 2). On day 7 of the cultivation period, 40% more FW was evident in the glucose/polysorbate 80-treated *H. erinaceus* (DAOMC 251029) relative to the additive-free cultures of this strain. Similarly, the combined glucose/polysorbate 80 treatment yielded at least 120% more mycelial FW in *H. americanum* (DAOMC 251011) and *H. erinaceus* (DAOMC 196448) relative to mycelia cultured on PDB alone. It is worth noting that the effect of the combined additive treatment was evident on day 7 and maintained thereafter in *H. erinaceus* (DAOMC 196448) but was not apparent until the 14th day of cultivation in *H. americanum* (DAOMC 251011). The benefit of the 1% (*w*/*v*) surfactant addition on the FW yield of the two *H. erinaceus* strains and *H. americanum* (DAOMC 251011) mirrors previous studies on *Lentinus edodes* [24] and *Cordyceps sinensis* [25]. There was no impact of supplemental glucose and the surfactant on the mycelial growth dynamics of *H. americanum* (DAOMC 21467) and *H. coralloides* (DAOMC 251017). For these two strains, the possibility remains that larger concentrations of polysorbate 80 are required to promote increased growth of their mycelia. In fact, Okumura et al. [23] determined that Tween 80 concentrations of 2–4% (*w*/*v*) are required to enhance mycelial growth of the *H. erinaceus* strain investigated in that study.

Generally, submerged mycelia of *H. erinaceus* tend to develop as spherical pellets approximating a diameter of 2 to 4 mm [23,35]. Similarly, mycelial pellets were apparent for the *Hericium* strains investigated in our study (Figure 2B). Moreover, the glucose/polysorbate 80-treated mycelia tended to be paler in color as compared to the other two treatments. Interestingly, pellets appeared finer and more dispersed in the *H. americanum* cultures after 14 days of submerged cultivation with polysorbate 80. A similar morphological change was observed in the medicinal mushroom *C. sinensis* when polysorbate 80 was applied to submerged cultures [25]. Although not assessed here, these phenomena likely coincide with a smaller mycelial pellet size relative to surfactant-free cultures, which is due to the surfactant’s inhibition of mycelial aggregation. In fact, Okumura et al. [23] reported that smaller mycelia diameters and more viscous cultures are formed with the addition of agar particles (i.e., between 0.1 and 0.3% *w*/*v*) to shake-flask cultures of *H. erinaceus*.

### 2.2. Effect of Polysorbate 80 on the Erinacine Profiles of Hericium spp. Strains

**Figure 3:**
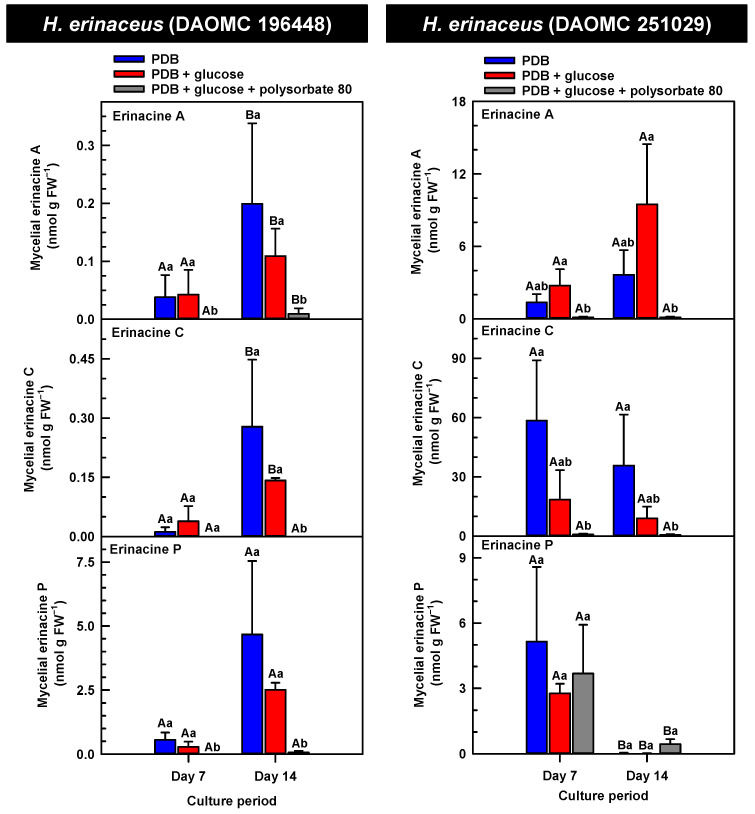
Alterations in the profiles of erinacines A, C, and P in the mycelial extracts of submerged *H. erinaceus* strains grown on potato dextrose broth (PDB) for up to 14 days. Within each plot, blue-filled bars, red-filled bars and grey-filled bars represent the respective erinacine concentration within mycelia cultivated on PDB, PDB supplemented with 2% (*w*/*v*) glucose, and PDB supplemented with 2% (*w*/*v*) glucose and 1% (*w*/*v*) polysorbate 80. Each datum represents the mean erinacine concentration ± SE of three separate experimental replicates. Within each plot, uppercase letters represent statistical comparisons within a cultivation treatment across the culture period. Lowercase letters represent statistical comparisons across the cultivation treatments at each culture period sampling time. Means sharing the same letter are not statistically different (*p* ≤ 0.05).

**Figure 4:**
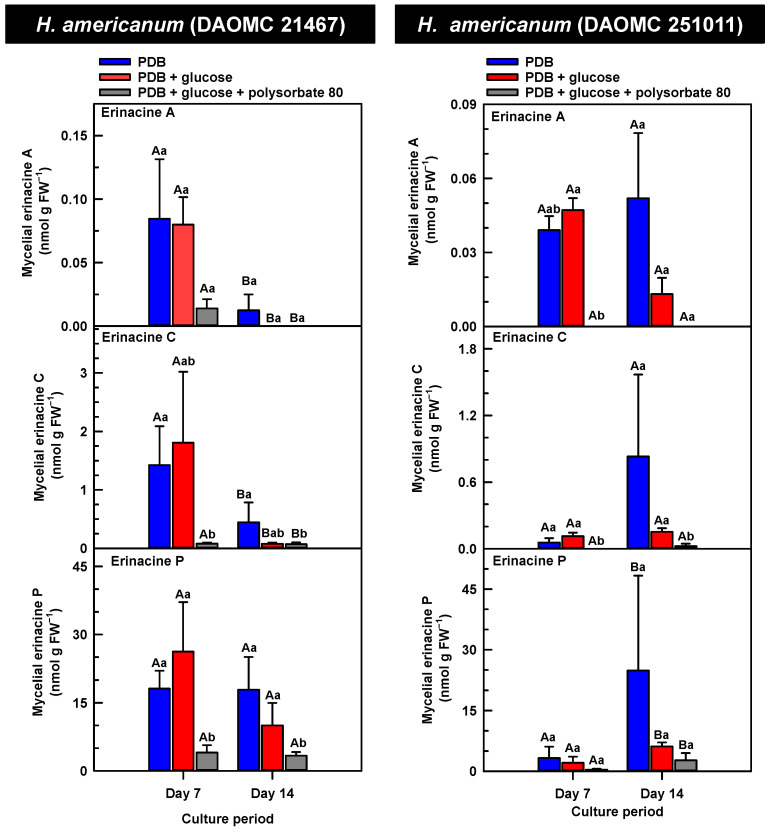
Alterations in the profiles of erinacines A, C, and P in the mycelial extracts of submerged *H. americanum* strains grown on potato dextrose broth (PDB) for up to 14 days. Within each plot, blue-filled bars, red-filled bars, and grey-filled bars represent the respective erinacine concentration within mycelia cultivated on PDB, PDB supplemented with 2% (*w*/*v*) glucose, and PDB supplemented with 2% (*w*/*v*) glucose and 1% (*w*/*v*) polysorbate 80. Each datum represents the mean erinacine concentration ± SE of three separate experimental replicates. Within each plot, uppercase letters represent statistical comparisons within a cultivation treatment across the culture period. Lowercase letters represent statistical comparisons across the cultivation treatments at each culture period sampling time. Means sharing the same letter are not statistically different (*p* ≤ 0.05).

**Figure 5:**
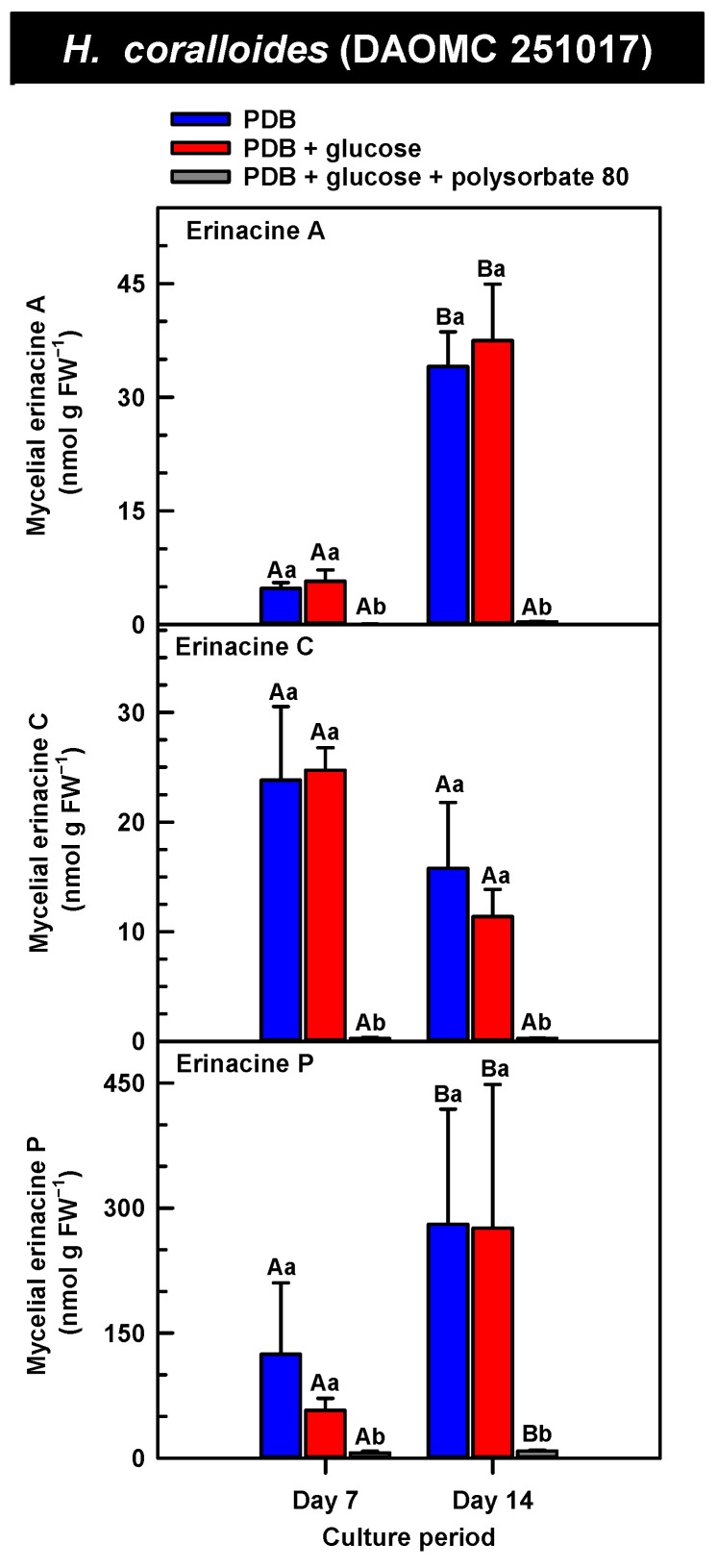
Alterations in the profiles of erinacines A, C, and P in the mycelial extracts of submerged *H. coralloides* (DAOMC 251017) grown on potato dextrose broth (PDB) for up to 14 days. Within each plot, blue-filled bars, red-filled bars, and grey-filled bars represent the respective erinacine concentration within mycelia cultivated on PDB, PDB supplemented with 2% (*w*/*v*) glucose, and PDB supplemented with 2% (*w*/*v*) glucose and 1% (*w*/*v*) polysorbate 80. Each datum represents the mean erinacine concentration ± SE of three separate experimental replicates. Within each plot, uppercase letters represent statistical comparisons within a cultivation treatment across the culture period. Lowercase letters represent statistical comparisons across the cultivation treatments at each culture period sampling time. Means sharing the same letter are not statistically different (*p* ≤ 0.05).

**Figure 6:**
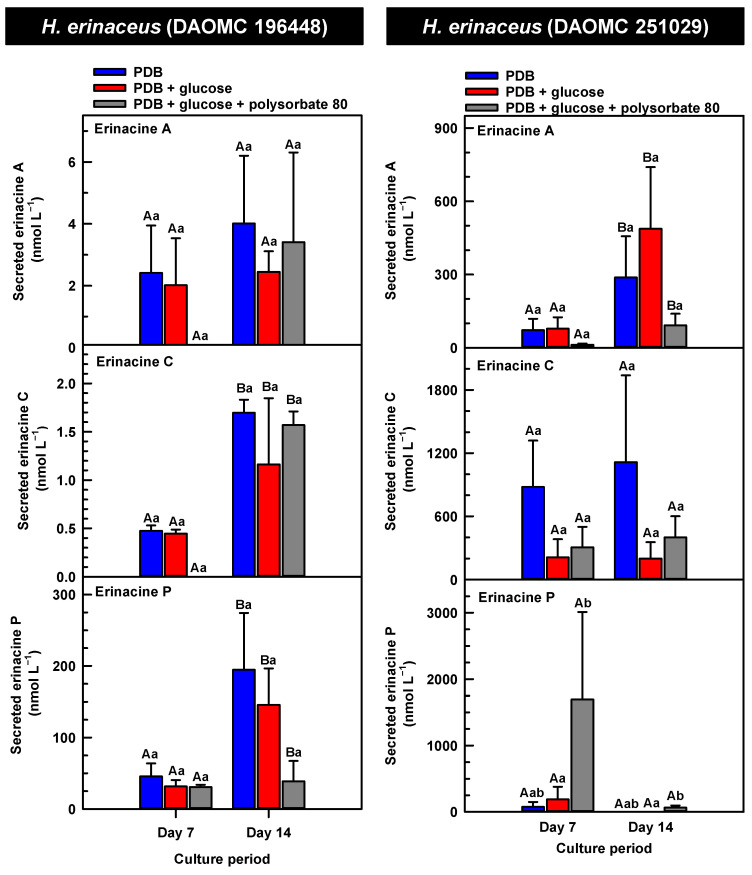
Alterations in the profiles of secreted erinacines A, C and P in the culture medium filtrate of submerged *H. erinaceus* strains grown on potato dextrose broth (PDB) for up to 14 days. Within each plot, blue-filled bars, red-filled bars, and grey-filled bars represent the respective erinacine concentration within mycelia cultivated on PDB, PDB supplemented with 2% (*w*/*v*) glucose, and PDB supplemented with 2% (*w*/*v*) glucose and 1% (*w*/*v*) polysorbate 80. Each datum represents the mean erinacine concentration ± SE of three separate experimental replicates. Within each plot, uppercase letters represent statistical comparisons within a cultivation treatment across the culture period. Lowercase letters represent statistical comparisons across the cultivation treatments at each culture period sampling time. Means sharing the same letter are not statistically different (*p* ≤ 0.05).

**Figure 7:**
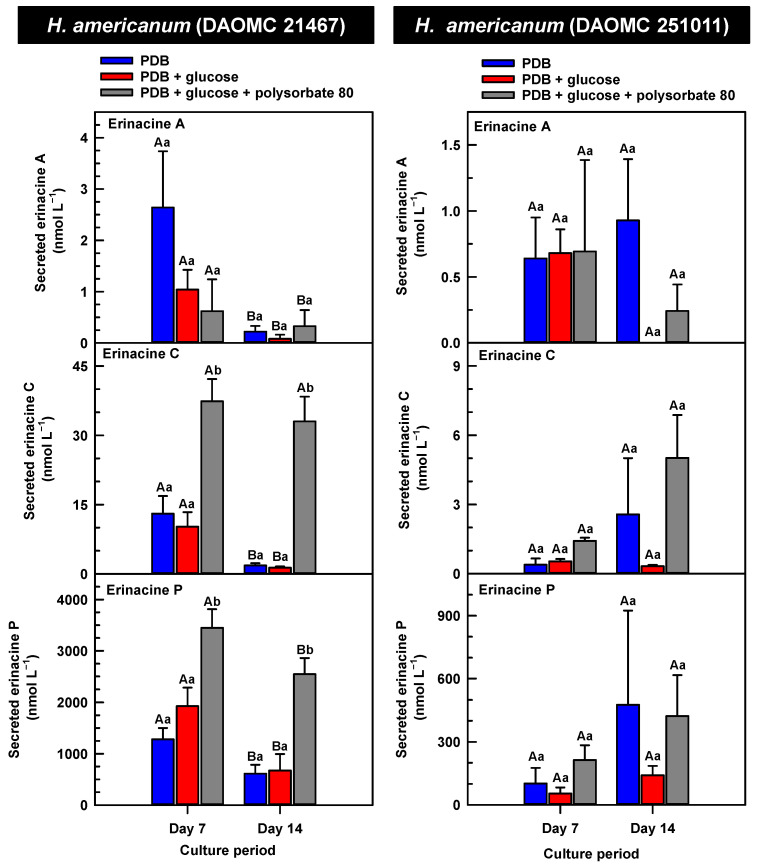
Alterations in the profiles of secreted erinacines A, C and P in the culture medium filtrate of submerged *H. americanum* strains grown on potato dextrose broth (PDB) for up to 14 days. Within each plot, blue-filled bars, red-filled bars and grey-filled bars represent the respective erinacine concentration within mycelia cultivated on PDB, PDB supplemented with 2% (*w*/*v*) glucose, and PDB supplemented with 2% (*w*/*v*) glucose and 1% (*w*/*v*) polysorbate 80. Each datum represents the mean erinacine concentration ± SE of three separate experimental replicates. Within each plot, uppercase letters represent statistical comparisons within a cultivation treatment across the culture period. Lowercase letters represent statistical comparisons across the cultivation treatments at each culture period sampling time. Means sharing the same letter are not statistically different (*p* ≤ 0.05).

**Figure 8:**
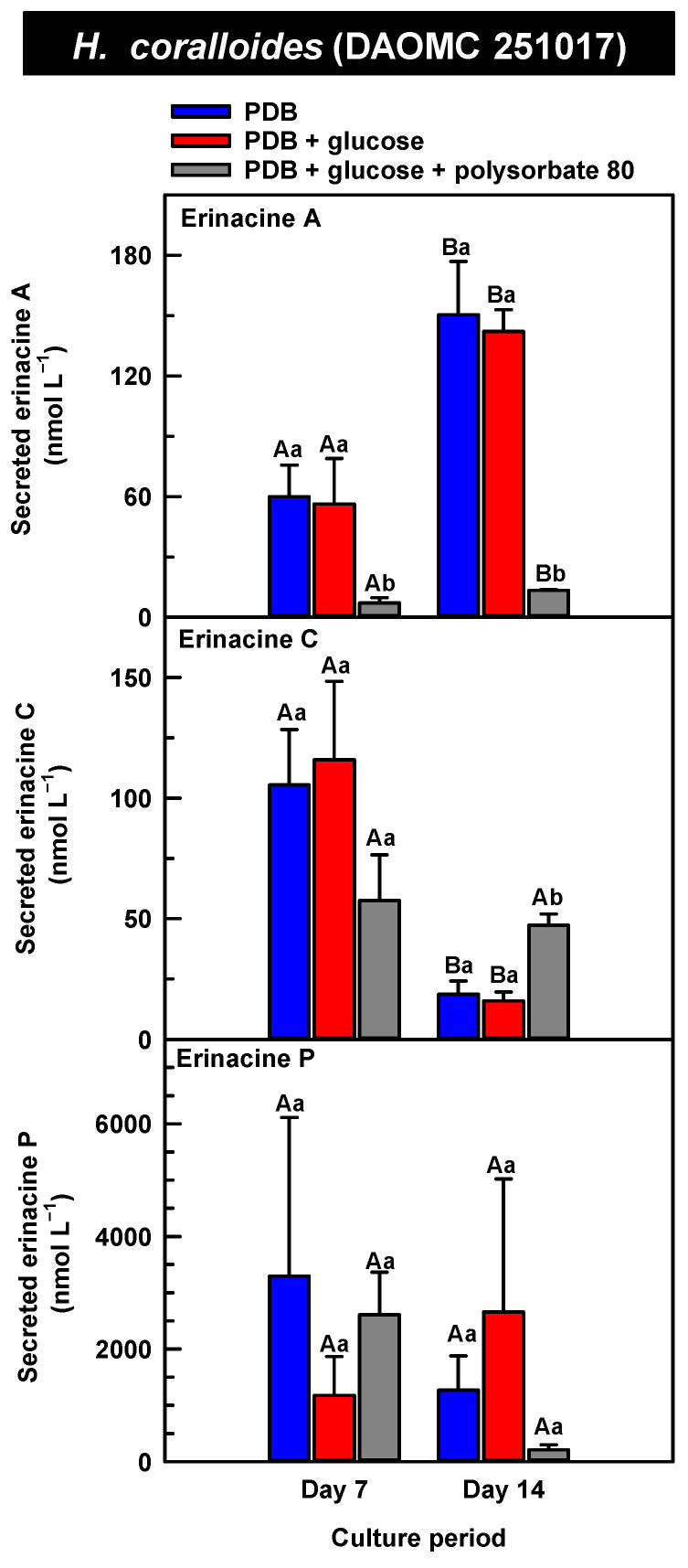
Alterations in the profiles of secreted erinacines A, C, and P in the culture medium filtrate of submerged *H. coralloides* (DAOMC 251017) grown on potato dextrose broth (PDB) for up to 14 days. Within each plot, blue-filled bars, red-filled bars, and grey-filled bars represent the respective erinacine concentration within mycelia cultivated on PDB, PDB supplemented with 2% (*w*/*v*) glucose, and PDB supplemented with 2% (*w*/*v*) glucose and 1% (*w*/*v*) polysorbate 80. Each datum represents the mean erinacine concentration ± SE of three separate experimental replicates. Within each plot, uppercase letters represent statistical comparisons within a cultivation treatment across the culture period. Lowercase letters represent statistical comparisons across the cultivation treatments at each culture period sampling time. Means sharing the same letter are not statistically different (*p* ≤ 0.05).

Ultra-performance liquid chromatography-electrospray ionization-tandem mass spectrometry (UPLC ESI-MS/MS) analysis was used to assess the impact of supplemental glucose and polysorbate 80 on the erinacine profiles of various *H. erinaceus*, *H. americanum* and *H. coralloides* strains. A specific emphasis was the quantification of the concentrations of erinacines A, C, and P within the mycelial extract. Secondly, we quantified the concentrations of the secreted form of these erinacines following their extraction from the culture medium filtrate of each *Hericium* shake-flask culture. To develop the UPLC-ESI-MS/MS method for erinacine analysis, ESI of these molecules was performed in both positive ion and negative ion modes. The negative ion mode was selected for the quantification analysis as it yielded greater ionization signal intensities for all erinacines tested. The negative ion MS/MS spectra of the authentic erinacine standards are provided in Figure S1 (Supplementary Information). All erinacine profiles as reported in Figure 3, Figure 4, Figure 5, Figure 6, Figure 7 and Figure 8 are based on the detection of peaks corresponding to the respective formic acid adduct ions ([M+HCOOH−H]^−^) of erinacine P (*m*/*z* 537.3, retention time = 12.7 min), erinacine A (*m*/*z* 477.3, retention time = 13.5 min), and erinacine C (*m*/*z* 479.3, retention time = 14.4 min). For all three erinacines, their formic acid adduct ions had much higher signal intensities and the linear calibration range was broader as compared to their respective [M−H]^−^ ions. As an example, a comparison of the respective signal intensities of the [M+HCOOH−H]^−^ and [M−H]^−^ of the authentic erinacine C standard is provided in Figure S2 (Supplementary Information). The smallest amount of the authentic standard of erinacine A that was detected using the [M+HCOOH−H]^−^ signal was 0.013 pmol. The smallest amount of the [M+HCOOH−H]^−^ signal that was detected for the authentic standards of erinacines C and P was 0.0196 pmol. By contrast, there was little detection of erinacines A, C, and P below the respective injections of 24 pmol, 10 pmol, and 40 pmol when using their [M−H]^−^ peaks. In positive mode, only the [M+H]^+^ signal of erinacine A was detected at the sub-pmol amounts that were analyzed for all three erinacines. It is worth noting that the formic acid adduct of erinacine A has been previously used to monitor the extraction and purification of this compound from *H. erinaceus* [36]. For the quantification of erinacines A, C, and P within each sample, [M+HCOOH−H]^−^ peak areas were compared to a known range of authentic erinacine standards eluting at the same retention times as described above. In this study we did not quantify other erinacines (e.g., erinacine B, erinacine S), due to the absence of commercial sources of their authentic standards, and lack of publicly available MS/MS fragmentation patterns.

*H. erinaceus* is a source of erinacine A, although its concentration within mycelia is strain specific [17]. Moreover, there is evidence that erinacines C and P occur in *H. erinaceus* mycelia grown under submergence [18]. Similarly, UPLC-ESI-MS/MS analysis revealed the presence of erinacines A, C, and P within the mycelial extracts of all five *Hericium* strains (Figure 3, Figure 4 and Figure 5). This included evidence for the occurrence of erinacines A, C, and P in North American strains of *H. americanum* and *H. coralloides* (Figure 4 and Figure 5). Liu et al. [17] reported high erinacine A concentrations in strains putatively identified as *H. coralloides*, but no genetic basis for the taxonomic annotation was provided. Moreover, Meng et al. [37] compared the relative levels of erinacines in mycelia and fruiting bodies of the *H. coralloides* strain ‘77’. Unfortunately, the Meng et al. study [37] compared the UV absorbance of the extract at 210 nm to a known concentration range of authentic baicalin. It is important to note that baicalin is a flavonoid and not a diterpenoid. In addition, the spectrophotometric analysis of a crude fungal extract at 210 nm will likely quantify many interfering non-erinacine compounds. To our knowledge, we are the first to report and quantify any erinacine metabolite originating from the mycelia of *H. coralloides* and *H. americanum*.

Overall, erinacine concentrations in *H. coralloides* were one to three orders of magnitude greater than those detected in the other strains (Figure 5). This difference was most apparent in cultures that were grown without media additives. We found that the alterations in the levels of erinacine A as a function of growth period were strain specific. In shake-flask cultures grown without media additives, the mycelial erinacine A concentration increased 4.2-fold and 6.1-fold by day 14 relative to day 7 of cultivation in *H. erinaceus* (DAOMC 196448) and *H. coralloides* (DAOMC 251017), respectively. By comparison, over the same period there was an 85% decrease in erinacine A concentrations of *H. americanum* (DAOMC 21467), and no change was apparent in the remaining two strains. A recent study determined that erinacine A concentrations ranged from 0.2 to 42 mg g lyophilized mycelia^−1^ across various *H. erinaceus* strains sourced from China [17]. Smaller erinacine A concentrations of 105 and 150 µg g^−1^ lyophilized mycelia have been reported for two *H. erinaceus* strains isolated from select deciduous forests within the Tuscany region of Italy [38]. Given that the water content of mycelia can be as much as 90% of the original FW [39], the erinacine A concentrations of the Corana et al. study [35] equate to 10.5–15 µg g FW^−1^. In our study, the peak erinacine A concentrations that were detected in the two *H. erinaceus* strains were 0.2 ± 0.14 and 9.5 ± 4.9 nmol g FW^−1^, which are equivalent to 0.09 ± 0.06 and 4.3 ± 2.1 µg g FW^−1^, respectively. The difference between the erinacine A profiles in the *H. erinaceus* strains evaluated in our study versus those of the previous studies [17,38] could be due to a myriad of factors including erinacine extraction methodologies. The yield of mycelial erinacines extracted by ethyl acetate in our study may have been greater with an alternate extraction solvent system. In fact, a recent study by Naumoska et al. [36] revealed that high-performance thin layer chromatography detected more erinacine A in *H. erinaceus* mycelia extracted with an aqueous mixture of 70% (*v*/*v*) ethyl acetate or 70% (*v*/*v*) ethanol relative to extracts prepared with either pure solvent.

Strain specific metabolite profiles were apparent for the mycelial concentrations of erinacines C and P with respect to culture period (Figure 3, Figure 4 and Figure 5). A 70% decline in erinacine C was apparent in *H. americanum* (DAOMC 21467) over the 14-day culture period. Conversely, a 22.5-fold increase in this metabolite was apparent in *H. erinaceus* (DAOMC 196448). Erinacine C concentrations in the other strains were unaffected by culture period. In control cultures, there was a 6.7-fold and 1.3-fold accumulation of erinacine P in *H. americanum* (DAOMC 251011) and the *H. coralloides* strain, respectively. Conversely, erinacine P peaked at day 7 of cultivation in *H. erinaceus* (DAOMC 251029) and declined by 99.5% thereafter. Erinacine P concentrations were stable throughout the culture period in the remaining strains. The accumulation pattern of the erinacines is likely a consequence of the degree of expression of key erinacine biosynthesis genes. It is expected that the accumulation of erinacine C is dependent upon the increased accumulation of *EriB* transcripts and protein levels. EriB is a NAD(P) oxidoreductase that catalyzes the conversion of erinacine Q to erinacine P, as well as the conversion of erinacine B to erinacine C (Figure 1). Erinacine A is also derived from erinacine B but the precise enzymatic steps governing this conversion are unknown. Generally, the expression of erinacine biosynthesis genes occurs in mycelia of *H. erinaceus*, but not its fruiting bodies [40]. Doar et al. [13] determined that many erinacine biosynthesis genes are expressed in *H. erinaceus* mycelia cultivated in shake-flasks, but their temporal gene expression patterns did not overlap with erinacine accumulation profiles. The previous study evaluated erinacine profiles and gene expression in mycelia that were harvested after 21 days of cultivation. Nonetheless it is worth noting that some degree of erinacine production can occur within the first week of cultivation as evidenced in our study and prior research [18]. Thus, future research should consider monitoring transcript profiles corresponding to erinacine biosynthesis genes as a function of *H. erinaceus* mycelial cultivation period. This can also be explored in *H. coralloides* given the recent sequencing and assembly of its genome, and more importantly the prediction of various putative genes encoding early and late erinacine biosynthesis steps [37].

The UPLC-ESI-MS/MS analysis of the mycelia extracts revealed there was no impact of supplemental glucose alone (i.e., without polysorbate 80) on the accumulation of erinacines A, C, and P, regardless of *Hericium* strain. Overall, the supplemental glucose trends described in our study contrast with the benefits of glucose (and other carbon sources such as starch) on the biomass yield and enhanced production of erinacine A in *H. erinaceus* shake-culture flasks [15]. The aforementioned study quantified erinacine A in mycelial cultures that were harvested after 28 days of growth, whereas we assessed erinacine profiles over the initial 14 days of cultivation. In addition, their study compared erinacine production in cultures grown on a basal medium with and without glucose, whereas our study compared cultures grown on PDB (a medium with a carbon source) with and without supplemental glucose.

Here we provide evidence that polysorbate 80 reduced the concentrations of mycelial erinacines on day 7 and/or day 14 of the culture period (Figure 3, Figure 4 and Figure 5), as well as end of culture period yields of erinacines (see Table S1, Supplementary Information). Polysorbate 80 reduced erinacine concentrations in *H. coralloides* by as much as 95–99% (Figure 5) as well as culture yields relative to the other cultivation treatments. The surfactant reduced erinacine A and C concentrations in *H. erinaceus* (DAOMC 251029) by a similar extent, although erinacine P concentrations were not affected. Similarly, a 95–100% reduction in erinacine concentrations was apparent in *H. erinaceus* (DAOMC 196448). In this *H. erinaceus* strain, the only exception was that mycelial erinacine C concentrations that were detected on day 7 were unaffected by media additives. Polysorbate 80 reduced the concentrations of erinacines C and P in *H. americanum* (DAOMC 21467) by as much as 83 to 95%, but there was no effect on erinacine A production. Finally, erinacines A and C concentrations were 97 to 100% lower in polysorbate 80-containing cultures of *H. americanum* (DAOMC 251011) compared to the other treatments. In this strain, the only exceptions were that polysorbate 80 had no effect on the concentration of erinacine A on day 14 and erinacine P concentrations at both sampling times of the culture period. If it is assumed that polysorbate 80 improved glucose uptake in the *Hericium* strains investigated here, the decrease in erinacine concentrations by polysorbate 80 is likely at the expense of a competing metabolic pathway such as exopolysaccharide (e.g., β-glucan) production. Exopolysaccharides are derived from nucleotide sugars (e.g., uridine diphosphate-glucose), which are formed from available sugar units such as glucose [41]. In fact, improvements in glucose uptake and exopolysaccharide production occur in mycelial cultures of *P. tuber-regium* and *H. erinaceus* supplemented with Tween 80 [20,23]. Similarly, the addition of Tween 80 increases exopolysaccharide production in *Lentinus tigrinus* [26] and *Cordyceps sinensis* [25].

The secreted forms of all the erinacines were collected from the culture medium filtrate following the harvest of mycelial pellets by vacuum filtration. For the most part, erinacines A, C, and P were present in the culture media filtrate of all five *Hericium* strains and their cultivation treatments (Figure 6, Figure 7 and Figure 8). The range of secreted erinacine A concentrations detected across all cultures ranged from 0 to 487 ± 253 nmol L^−1^ (i.e., 0 to 210 ± 109 µg L^−1^ equivalence). To our knowledge, this is the first report of the secretion of erinacine A into the medium of *Hericium* mycelia. The concentration of secreted erinacine C ranged from 0 to 1115 ± 824 nmol L^−1^ (i.e., 0 to 0.48 ± 0.36 mg L^−1^ equivalence). There were only a few exceptions where erinacines were not detected. This included the absence of erinacine A in the glucose-supplemented culture of *H. americanum* (DAOMC 251011) on day 14 of cultivation, and the absence of both erinacines A and C in the glucose/polysorbate 80 co-treated culture of *H. erinaceus* (DAOMC 196448) on day 7 of cultivation (Figure 6 and Figure 7). Finally, the secreted erinacine P detected across all *Hericium* cultures ranged from 0.173 ± 0.173 to 3450 ± 361 nmol L^−1^ (i.e., 0.1 ± 0.1 µg L^−1^ to 1.7 ± 0.18 mg L^−1^ equivalence). The maximum concentration of secreted erinacine C detected in this study is one to two orders of magnitude smaller than the erinacine C concentrations reported for shake-flask cultures of *H. erinaceus* (FU70034) grown on yeast-salt medium supplemented with and without various carbon sources including the optimal source oatmeal [18]. Similarly, the concentration of erinacine P detected in this study is two orders of magnitude lower than the Shen et al. [18] study. It is worth noting that this previous publication used high-performance liquid chromatography-diode array detection (HPLC-DAD) at 210 nm, but the precise retention times and the quantification method for erinacines C and P peaks as resolved from the HPLC-DAD analysis were not described.

We also evaluated the changes in the concentrations of the secreted forms of erinacines A, C, and P across all *Hericium* strains as a function of cultivation period and the additive treatments. Overall, the accumulation patterns of the secreted erinacines were specific to each *Hericium* strain. In *H. erinaceus* (DAOMC 196448), as much as 2.5-fold and 3.6-fold more of the secreted forms of erinacines C and P, respectively, were apparent in the culture medium filtrate that was collected on day 14 relative to day 7 of cultivation (Figure 6). The secreted erinacine A profiles were stable throughout the cultivation period. There was no impact of supplemental glucose and polysorbate 80 on the secreted erinacine profiles in this strain. To a similar extent, the secreted erinacine profiles in *H. americanum* (DAOMC 251011) were stable with culture period and unaffected by media additives (Figure 7). Polysorbate 80 impacted the concentrations of the secreted erinacines in the shake-flask cultures of the other three strains. In *H. americanum* (DAOMC 21467), the secreted erinacine A concentrations declined with culture period, regardless of culture treatment. The concentrations of secreted erinacines declined by 53–92% over the 14-day cultivation period in polysorbate 80-free cultures of this strain, whereas the secreted erinacines P and C concentrations were stable up until day 14 yielding up to 3.2-fold and 24-fold more, respectively, than the other two treatments. In *H. erinaceus* (DAOMC 251029), only erinacine A profiles were altered by the cultivation period, with as much as 5.2-fold greater concentrations at day 14 relative to day 7. Interestingly, polysorbate 80 increased the concentration of secreted erinacine P by one to two orders of magnitude. Finally, the profiles of secreted erinacines A, C and P in the *H. coralloides* shake-flask cultures were variably affected by cultivation period and treatment (Figure 8). For example, secreted erinacine A concentrations were 87–91% smaller in polysorbate 80-containing cultures than the other two treatments. Although secreted erinacine C concentrations tended to decline with culture period, no change was apparent in polysorbate 80-containing cultures. Thus, by day 14 of cultivation there was up to 2-fold more erinacine C in *H. coralloides* shake-flask cultures containing polysorbate 80 than the other treatments. Secreted erinacine P profiles were stable in *H. coralloides* and unaffected by supplemental glucose and polysorbate 80. Polysorbate 80 and supplemental glucose had no effect on the secreted erinacine profiles in *H. erinaceus* (DAOMC 196448) and *H. americanum* (DAOMC 251011). 

The negligible effect of polysorbate 80 on the profiles of secreted erinacines A, C, and P in *H. erinaceus* (DAOMC 196448) and *H. americanum* (DAOMC 251011) may be due to limited membrane leakage of intracellular metabolites to the culture broth and/or decreased erinacine export activity. Alternatively, polysorbate 80 may promote increased membrane leakage and/or erinacine export and, hence, greater secretion of erinacines A, C, and P in *H. erinaceus* (DAOMC 251029), *H. americanum* (DAOMC 21467), and *H. coralloides* (DAOMC 251017). Thus far, it is known that the high concentrations of Tween 80 enhance membrane permeability in submerged mycelia [20,21]. The membrane permeability is associated with increased reactive oxygen species concentrations and alterations in membrane lipid constituents, and it has been proposed that the membrane lipids profiles incorporate oleic acid derived from the surfactant molecules [20,21], but there is no direct evidence to support this. It remains to be determined whether the *Hericium* strains investigated in this study vary in their membrane permeability to polysorbate 80. By contrast the mechanism for the alteration of membrane lipid profiles with surfactant addition is likely due to increased fatty acid desaturase activity. In fact, Lei et al. [42] reported that the surfactant Triton X-100 enhanced the secretion of the pigment hypocrellin A in *Shiraia bambusicola* mycelia, which was correlated with the increased transcription of several fatty acid desaturases genes. This same study found that Triton X-100 increased the expression of genes encoding for major facilitator superfamily transporters and ATP-binding cassette transporters. Similarly, submerged cultivation of the saprophytic mold *Aureobasidium pullulans* with Tween 80 enhanced the production of the biopolymer pullulan [43]; this phenomenon coincided with the increased gene expression of a transmembrane protein. Interestingly the ATP-binding cassette transporter gene *EriD* is within a gene cluster with other erinacine biosynthesis genes in the *H. erinaceus* genome [13]. Similarly, a putative ATP-binding cassette transporter (i.e., *Her011460*) occurs within an erinacine biosynthesis gene cluster on chromosome 13 of *H. coralloides* [37]. It is tempting to speculate that erinacines A, C and P in the *H. erinaceus* strains investigated here are secreted through EriD transporters into the culture broth. Similarly, erinacine secretion from *H. coralloides* may involve a transporter encoded by *Her011460*. A transcriptomics analysis is required to assess whether transporter gene expression is altered in mycelia treated with polysorbate 80.The biological relevance of erinacine secretion from mycelia is unknown. One possibility is that erinacines are secreted as communication signals with their environment. In the mycelia of other fungi, secretion of secondary metabolites appears critical for various reasons including outcompeting other microbes, protection against insects, or promoting sporulation in other fungi that are in proximity [44]. Given that *Hericium* spp. are wood-rot fungi, it is tempting to speculate that secreted erinacines facilitate the colonization of decaying wood of hardwood trees that serve as the natural growth substrate for these mushrooms.

## 3. Materials and Methods

### 3.1. Fungal and Chemical Materials

*Hericium* cultures were attained from the Canadian Collection of Fungal Cultures. This included two *H. erinaceus* strains DAOMC 196448 and DAOMC 251029, two *H. americanum* strains DAOMC 251011 and DAOMC 21467, and *H. coralloides* strain DAOMC 251017. Genetic sequencing of these strains was performed at the Agriculture and Food Laboratory at the University of Guelph. Briefly, for each strain genomic DNA was isolated from 21-day old mycelia harvested from PDA plates and PCR-amplified using standard molecular biology procedures. The size of each of the amplification products corresponding to the ITS and large subunit (LSU) regions of ribosomal DNA were confirmed by gel electrophoresis prior to purification and sequencing on an Applied Biosystems ABI 3730XL Genetic Analyzer (Life Technologies, Burlington, ON, Canada). The ITS and LSU sequences were analyzed with ABI Prism^TM^ Sequencing Analysis software (version 7) and compared to publicly available ITS and LSU sequences deposited to the NCBI database (https://www.ncbi.nlm.nih.gov/; see Table S2, Supplementary Information). Unless otherwise mentioned, all chemicals were purchased from Millipore-Sigma Canada (Oakville, ON, Canada). HPLC grade solvents were from Fisher Scientific Canada (Mississauga, ON, Canada).

### 3.2. Mycelia Cultivation

For each *Hericium* strain, the general approach used for mycelia subculturing, seed culture preparation, homogenization, and inoculation of shake-flask liquid cultures was adapted from previous methods [18,23]. Briefly, the mycelia of each *Hericium* strain were separately propagated on PDA plates for 21 days in the dark at 24 °C. PDA was prepared with 24 g L^−1^ PDB (BioShop, Burlington, ON, Canada) and 15 g L^−1^ agar. Thereafter, a 1 cm^2^ agar disc was excised from the growing edge of mycelial growth and the inoculum transferred to a fresh PDA plate. After 21 subsequent days, seed cultures for each strain were prepared in 250 mL Erlenmeyer flasks containing 50 mL of 24 g L^−1^ PDB. For this each culture flask contained an inoculum of 5 agar discs, with each disc measuring 10 mm diameter. The seed cultures of mycelia were transferred to an Excella E25 Incubator Shaker (Fisher Scientific, Ottawa, ON, Canada) and shaken at 120 rpm at 25 °C under darkness for 7 days.

The seed cultures were used to prepare experimental culture flasks for the investigation of the impact of glucose and polysorbate 80 on the erinacine profiles of *Hericium* germplasm cultivated in liquid shaking cultures. For each *Hericium* strain, 100 mL of seed culture was homogenized by applying up to five separate low intensity pulses of five seconds each with a sterilized immersion blender. Six separate 250 mL Erlenmeyer flasks containing up to 45 mL of PDB (final concentration of 24 g L^−1^) were each inoculated with a 5 mL aliquot of the homogenized seed culture. Four of the six flasks contained 2 % (*w*/*v*) glucose; two of these flasks contained 1% (*w*/*v*) polysorbate 80 (Fisher Scientific Canada, Mississauga, ON, Canada). To achieve these final concentrations, glucose (20% *w*/*v*) and polysorbate 80 (10% *w*/*v*) were each passed through separate sterile 0.2 µm syringe filters prior to their addition to flasks containing sterile PDB. The six flasks of each of the five *Hericium* species/strains were shaken at 120 rpm at 25 °C for up to 14 days. For each *Hericium* strain, one shake-flask culture for each of three cultivation treatments was harvested on day 7 of the experiment. The remaining shake-flask cultures containing glucose and cultures containing PDB alone were respectively supplied with an additional 2% (*w*/*v*) of glucose and an equivalent volume of sterile water on the following day and then harvested on day 14 of the experiment. Mycelia from each shake-flask culture were separated from the spent culture media by vacuum filtration through Whatman 2 filter paper that was placed over a Büchner funnel. The culture medium filtrate was extracted with ethyl acetate and analyzed by UPLC-ESI-MS/MS as described in Section 3.3 and Section 3.4. The mycelia were washed with Milli-Q processed water until the vacuum filtration flow-through was clear. The fresh mass of the moisture-free mycelia was taken and then flash frozen in liquid N_2_ and then held at −80 °C until required for erinacine analysis. The liquid cultivation of mycelia with or without additional glucose and polysorbate 80 was performed in triplicate.

### 3.3. Erinacine Extraction

The culture medium filtrate collected from the mycelia harvest of each species/strain cultivation treatment replicate was used for erinacine analysis. Erinacines within the culture medium filtrate were extracted with ethyl acetate as described by Shen et al. [18], with a few modifications. Briefly, a 20 mL aliquot of the culture medium filtrate was combined with an equal volume of HPLC-grade ethyl acetate and then shaken by vortex for 20 min. The ethyl acetate layer was collected after centrifugation (i.e., 1950× *g* for 30 s), and then dried by anhydrous sodium sulfate (Fisher Scientific Canada, Mississauga, ON, Canada). Equivalent volumes of the dried ethyl acetate layer were transferred into two separate conical tubes and dried under a stream of nitrogen gas. All dried extracts were frozen in liquid nitrogen and stored at −80 °C. One of the vials was used for UPLC-ESI-MS/MS analysis as described in Section 3.4. For each *Hericium* strain cultivation treatment replicate, the frozen mycelia were powdered under liquid N_2_ with a pre-chilled mortar and pestle. The extraction of mycelial erinacines was as described previously [18], with a few modifications. Briefly, a 100 mg subsample of the frozen mycelial powder was transferred to a previously frozen microfuge tube and combined with 5 vol of HPLC-grade ethyl acetate, and then vortexed for 20 min. The ethyl acetate extract was centrifuged for 30 s at 10,000× *g*, and the supernatant was withdrawn. The pellet residue was successively re-extracted twice, and the supernatants of all three extracts were pooled and dried under a stream of nitrogen gas. The dried residue was flash frozen in liquid N_2_ and transferred to a −80 °C freezer until required for UPLC-ESI-MS/MS analysis.

### 3.4. UPLC-ESI-MS/MS Analysis

Each vial containing the dried mycelial extract residue or the culture medium filtrate extract residue was resuspended in 200 µL of HPLC-grade methanol containing 2 µM of the diterpenoid gibberellic acid as an internal standard. The resuspended extract was filtered through a 0.45 µm Restek polytetrafluoroethylene syringe filter (Fisher Scientific Canada, Mississauga, ON, Canada) prior to UPLC-ESI-MS/MS analysis. For UPLC-ESI-MS/MS analysis, a 2 µL aliquot of the resuspended mycelial extract or culture medium filtrate extract was injected onto a Kinetex XB-C18 100 Å column (100 mm × 4.6 mm, 2.6 µm, Phenomenex Inc., Torrance, CA, USA) at a flow rate of 0.7 mL min^−1^. The column was connected to a Vanquish™ Flex Binary UPLC System (Waltham, MA, USA), and the temperature was maintained at 30 °C. The binary mobile phase consisted of solvent A (99.9% Milli-Q water/0.1% formic acid, *v*/*v*) and solvent B (94.9% methanol/5% acetonitrile/0.1% formic acid, *v*/*v*/*v*). The following solvent gradient was used: 0–8 min, 5% to 70% B; 8–15 min, 70% to 100% B; 15–20 min, 100% B; 20–24 min, 100% to 5% B; 24–28 min, 5% B. The samples were then analyzed with an in-line Thermo^®^ Scientific Q-Exactive™ Orbitrap mass spectrometer (Waltham, MA, USA). The mass spectrometer was operated in both positive and negative electrospray ionization modes. The spray voltage was set at 3.6 kV and 4.5 kV for negative and positive ion modes, respectively. All quantification was performed with signals generated in negative ion mode. MS data was collected using Full-MS/DDMS2 (TopN = 10) method, with normalized collision energy set at 30 and an intensity threshold of 1.0 × 10^5^ counts. Data was visualized and analyzed using Thermo FreeStyle™ 1.7 software. The annotation of eluted erinacines was based on the comparison of their retention times to those of authentic standards. Erinacine A was purchased from BOC Sciences (Shirley, NY, USA.). Erinacines C and P were purchased from Med Chem Express (Monmouth Junction, NJ, USA). To determine the concentration of erinacine A within each mycelial extract or culture media filtrate, the corresponding MS peak area of the [M+HCOOH−H]^−^ ion peak area following UPLC resolution of the extract or dilutions thereof was compared to a known range (0.013 to 80 pmol) of the authentic standard. For the quantification of each of erinacine C and erinacine P in each sample, the MS peak area of their respective [M+HCOOH−H]^−^ ion was compared to a known range (0.0196 to 80 pmol) of the respective authentic standard. The identification and quantification of other erinacines were not performed, as authentic standards are not commercially available and there is an absence of publicly available information for respective MS/MS fragmentation data.

### 3.5. Statistical Analysis

The statistical analysis of the impact of cultivation period and shake-flask additive treatment on mycelia biomass and erinacine concentrations was performed with R version 4.4.2 [45]. For all data sets, the Shapiro–Wilk test and the Levene’s test were used to, respectively, test for assumptions of normality and homoscedasticity. When necessary, a logarithmic transformation, with 0.0001 added to data sets containing zero, or a square root transformation was applied to ensure data satisfied assumptions of normality and homoscedasticity. To assess the impact of cultivation period and shake-flask culture additive treatments, a two-way ANOVA was used to analyze the data. For each data set, means were compared with a post hoc analysis using Tukey’s HSD (*p* ≤ 0.05). For data that did not satisfy normality assumptions after mathematical transformations, a non-parametric Aligned Rank Test was performed with the ARTool package 0.11.2 [46], and statistical significance was determined by contrast analysis [47].

## 4. Conclusions

Our study determined that mycelial and secreted forms of erinacines A, C, and P occur in various *Hericium* strains. We are the first to provide robust UPLC-ESI-MS/MS evidence for these erinacines in North American strains of *H. americanum* and *H. coralloides*. Moreover, the metabolite profiling analysis established *H. coralloides* (DAOMC 251017) as a promising source of erinacines, specifically erinacine P. The 14-day shake-flask cultivation period and procedure described in this study (i.e., in the absence of polysorbate 80) provides a strategy to produce *Hericium* extracts enriched in erinacine P, including from *H. coralloides* (DAOMC 251017). The information from our study provides the tools for assessing whether this erinacine biosynthesis intermediate has an impact on neurological-related processes, including those associated with diseases such as Alzheimer’s disease. There is a strong possibility that other erinacine pathway intermediates (e.g., erinacine Q) and end-products (e.g., erinacine B) occur in one or more of the *Hericium* strains. Additional research is required to determine whether these other erinacine end-products accumulate in one or more of the *Hericium* strains that we investigated. For many of the *Hericium* strains, erinacine production within the mycelia and secretion to the culture medium broth were repressed by polysorbate 80 addition to the cultivation medium. Transcriptomics and/or proteomics studies are required to assess whether structural and regulatory processes of the erinacine biosynthesis pathway in *H. erinaceus*, *H. americanum,* and *H. coralloides* strains are differentially regulated by polysorbate 80. Moreover, similar approaches could be used to determine whether erinacine biosynthesis mechanisms are differentially altered in the high-erinacine producing *H. coralloides* relative to the other *Hericium* strains. There was a differential impact of polysorbate 80 on the distribution of some secreted erinacines in a few of the *Hericium* strains. This included the increased accumulation of secreted erinacines C and P in *H. americanum* (DAOMC 21467) and secreted erinacine P in *H. erinaceus* (DAOMC 251029). These diverse erinacine production patterns are likely a consequence of differential regulation of gene expression and/or activities associated with erinacine export steps.

## Data Availability

The data presented in this study are available in the results section of this article.

## Supplementary Materials

The following supporting information can be downloaded at: https://www.mdpi.com/article/10.3390/molecules30132823/s1, Figure S1: Negative ion UPLC-ESI-MS/MS spectra of the authentic standards of erinacines A, C and P; Figure S2: Representative extracted ion chromatograms of the negative ion ([M−H]^−^) and formic acid adduct ion ([M+HCOOH−H]^−^) of a 40 pmol injection of an authentic erinacine C standard; Table S1: Impact of glucose (G) and polysorbate 80 (P80) on the yields of erinacines isolated from the mycelia of various *Hericium* strains grown in shake-flask cultures containing potato dextrose broth (PDB) for 14 days; Table S2: BLAST (2.16.0) analyses of PCR-amplification products of the internal transcribed spacer (ITS) region of the nuclear ribosomal DNA and the large subunit (LSU) ribosomal RNA gene from various *Hericium* strains.

## Abbreviations

The following abbreviations are used in this manuscript:

DAOMC	Canadian Collection of Fungal Cultures
PDA	Potato dextrose agar
PDB	Potato dextrose broth
UPLC-ESI-MS/MS	Ultra-performance liquid chromatography-electrospray ionization-tandem mass spectrometry
