# Supplementary material for: Polysorbate 80 Differentially Impacts Erinacine Production Profiles in Submerged Cultures of Hericium
Source: Molecules. 2025 Jun 30;30(13):2823. doi: 10.3390/molecules30132823 (PMC12251483; doi:10.3390/molecules30132823)
Supplement: Supplementary file 1 [file molecules-30-02823-s001.zip › molecules-3694475-supplementary.pdf]

Polysorbate 80 differentially impacts erinacine production profiles in submerged cultures of *Hericium*

Abigail Smith [1], Honghui Zhu [2], Lili Mats [2], and Gale Bozzo [1]*

[1] Department of Plant Agriculture, University of Guelph, 50 Stone Road East, Guelph, ON N1G 2W1, Canada

[2] Guelph Research and Development Centre, Agriculture and Agri-Food Canada, 93 Stone Road West, Guelph, ON N1G 5C9, Canada
* Correspondence: gbozzo@uoguelph.ca

Figure S1. Negative ion UPLC-ESI-MS/MS spectra of the authentic standards of erinacines A, C and P.

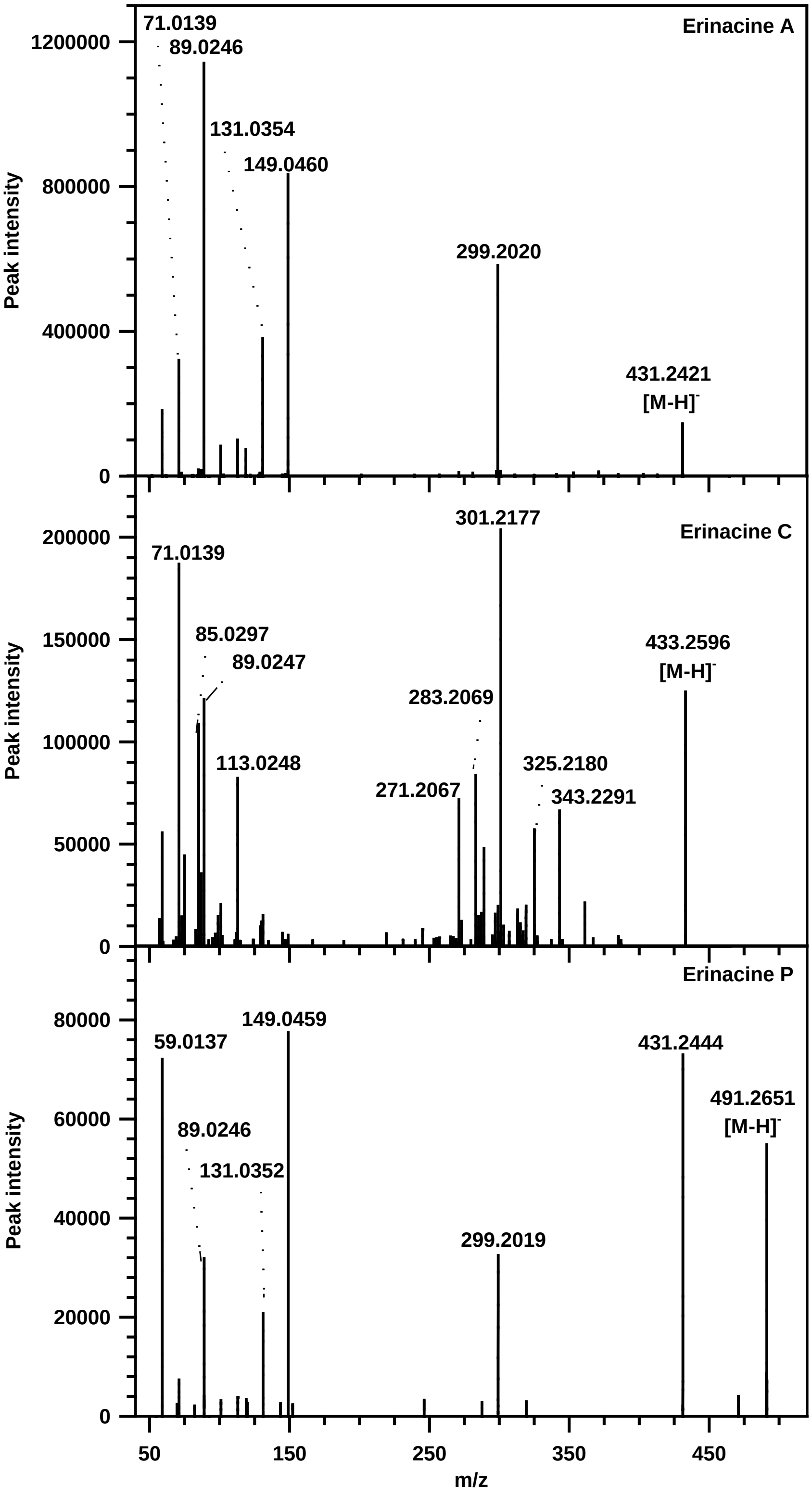

Figure S2. Representative extracted ion chromatograms of the negative ion ([M-H]$^-$) and formic acid adduct ion ([M+HCOOH-H]$^-$) of a 40 pmol injection of an authentic erinacine C standard.

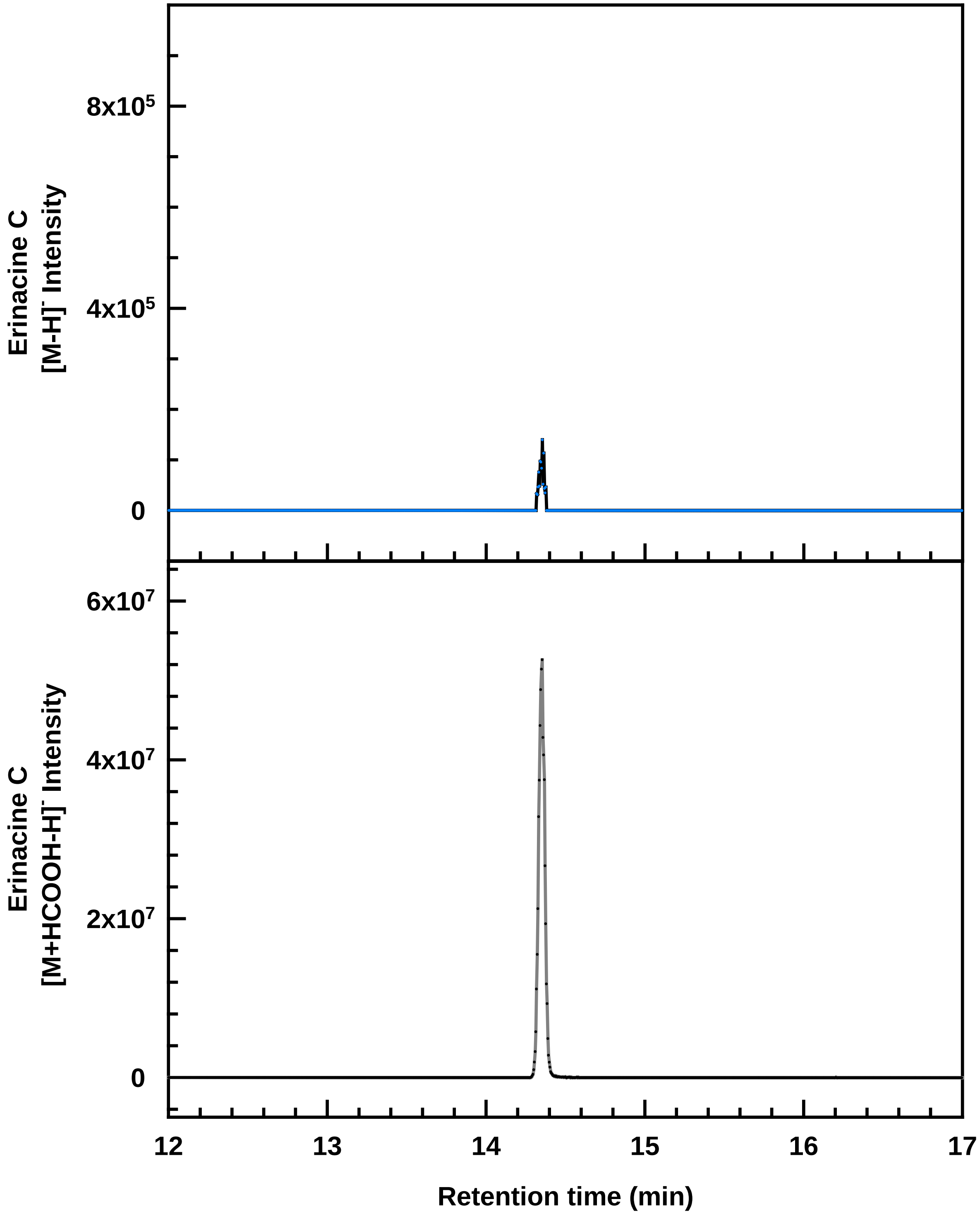

Table S1. Impact of glucose (G) and polysorbate 80 (P80) on the yields of erinacines isolated from the mycelia of various *Hericium* strains grown in shake-flask cultures containing potato dextrose broth (PDB) for 14 days

Strain	Cultivation treatment	Mycelia erinacine A (nmol L^{-1})*	Mycelia erinacine C (nmol L^{-1})*	Mycelia erinacine P (nmol L^{-1})*
H. erinaceus (DAOMC 196448)	PDB	1.25	1.74	29.22
	PDB +G	0.73	0.99	16.68
	PDB + G + P80	0.19	0	1.29
H. erinaceus (DAOMC 251029)	PDB	84.58	829.8	0.6
	PDB +G	177.12	168.1	0.3
	PDB + G + P80	2.49	11.6	8.8
H. americanum (DAOMC 21467)	PDB	0.16	5.88	236.23
	PDB +G	0	1.62	211.61
	PDB + G + P80	0	1.75	81.66
H. americanum (DAOMC 251011)	PDB	0.52	8.32	249.33
	PDB +G	0.17	2.00	81.0
	PDB + G + P80	0	0.44	53.1
H. coralloides (DAOMC 251017)	PDB	383.66	178.02	3161.11
	PDB +G	420.39	127.82	3097.55
	PDB + G + P80	3.68	2.75	88.87

*Calculated by multiplying the mean erinacine concentration expressed as nmol g fresh weight^{-1} (from data available in Figures 3, 4 and 5 of the manuscript) by the corresponding mean mycelia biomass expressed as g L^{-1} (from data available in Figure 2 of the manuscript).

Table S2. BLAST (2.16.0) analyses of PCR-amplification products of the internal transcribed spacer (ITS) region of the nuclear ribosomal DNA and the large subunit (LSU) ribosomal RNA gene from various *Hericium* strains.

Species	Strain identifier	Origin	Similarity % by ITS	Similarity % by LSU
H. americanum	DAOMC251011	Pennsylvania	100.0% - *H. americanum* HA2 (OR793914)*<br>99.8% - *H. americanum* HA11 (OR793921)<br>99.7% - *H. alpestre* DAOMC172297 (OR793952)	100% - *H. flagellum* voucher F314 (OR602364)<br>100% - *H. alpestre* DSM 108284 (MK491173)<br>99.8% - *H. americanum* AFTOL-ID 469 (DQ411538)
H. americanum	DAOMC21467	Ontario	100% - *H. americanum* HA11 (OR793921)<br>99.8% - *H. americanum* HA2 (OR793914)<br>99.84% - *H. alpestre* DAOMC172297 (OR793952)<br>99.7% - *H. flagellum* voucher G.M. 2014-10-12.2 (OM350398)<br>99.7% - *H. erinaceus* voucher DAOM_196728 (PP786303)	99.8% - *H. erinaceus* voucher Cui 16153 (MH085967)<br>99.7% - *H. americanum* (AF506458)<br>99.7% - *H. alpestre* DSM 108284 (MK491173)<br>99.7% - *H. flagellum* voucher G.M. 2014-10-12.2 (OM350398)
H. erinaceus	DAOMC196448	Virginia	99.3% - *H. erinaceus* CBS 485.95 (AY534583)<br>99.3% - *H. erinaceus* voucher TVR:3 (MT731944)	99.9% - *H. erinaceus* voucher DAOM/19644 (JN649345)<br>99.8% - *H. erinaceus* voucher Zh001 (KY655904)
H. erinaceus	DAOMC251029	Pennsylvania	100% - *H. erinaceus* DAOMC251029 (OR793939)<br>99.4% - *H. erinaceus* DAOMC251033 (OR793941)<br>99 % - *H. rajendrae*	100 % - *H. erinaceus* voucher DAOM/19644 (JN649345)<br>99.9% - *H. erinaceus* voucher Zh001 (KY655904)
H. coralloides	DAOMC251017	Virginia	99.4% - *H. coralloides* (MT759716)<br>99.2% - *H. coralloides* strain HC2 (OR793924)	100 % *H. coralloides* voucher Cui 14825 (MH085962)<br>99.8% *H. coralloides* (AF506459)

*GenBank nucleotide accession numbers are provided in parentheses.
